# Allele frequencies of hemojuvelin gene (*HJV*) I222N and G320V missense mutations in white and African American subjects from the general Alabama population

**DOI:** 10.1186/1471-2350-5-29

**Published:** 2004-12-20

**Authors:** James C Barton, Charles A Rivers, Sandrine Niyongere, Sean B Bohannon, Ronald T Acton

**Affiliations:** 1 Southern Iron Disorders Center, Birmingham, Alabama; 2 Department of Medicine, University of Alabama at Birmingham, Birmingham, Alabama; 3 Immunogenetics Program and Departments of Microbiology and Epidemiology and International Health, University of Alabama at Birmingham, Birmingham, Alabama

## Abstract

**Background:**

Homozygosity or compound heterozygosity for coding region mutations of the hemojuvelin gene (*HJV*) in whites is a cause of early age-of-onset iron overload (juvenile hemochromatosis), and of hemochromatosis phenotypes in some young or middle-aged adults. *HJV *coding region mutations have also been identified recently in African American primary iron overload and control subjects. Primary iron overload unexplained by typical hemochromatosis-associated *HFE *genotypes is common in white and black adults in Alabama, and *HJV *I222N and G320V were detected in a white Alabama juvenile hemochromatosis index patient. Thus, we estimated the frequency of the *HJV *missense mutations I222N and G320V in adult whites and African Americans from Alabama general population convenience samples.

**Methods:**

We evaluated the genomic DNA of 241 Alabama white and 124 African American adults who reported no history of hemochromatosis or iron overload to detect HJV missense mutations I222N and G320V using polymerase chain reaction-restriction fragment length polymorphism (PCR-RFLP) technique. Analysis for HJV I222N was performed in 240 whites and 124 African Americans. Analysis for HJV G320V was performed in 241 whites and 118 African Americans.

**Results:**

One of 240 white control subjects was heterozygous for *HJV *I222N; she was also heterozygous for *HFE *C282Y, but had normal serum iron measures and bone marrow iron stores. *HJV *I222N was not detected in 124 African American subjects. *HJV *G320V was not detected in 241 white or 118 African American subjects.

**Conclusions:**

*HJV *I222N and G320V are probably uncommon causes or modifiers of primary iron overload in adult whites and African Americans in Alabama. Double heterozygosity for *HJV *I222N and *HFE *C282Y may not promote increased iron absorption.

## Background

Homozygosity or compound heterozygosity for coding region mutations of the hemojuvelin gene (*HJV*) in whites is a cause of early age-of-onset iron overload (juvenile hemochromatosis), and of hemochromatosis phenotypes in some young or middle-aged adults [1-4]. *HJV *coding region mutations have also been identified recently in African American primary iron overload and control subjects [5]. We estimated the frequency of the *HJV *missense mutations I222N and G320V in Alabama white and African American adults who reported no history of hemochromatosis or iron overload, because primary iron overload unexplained by typical hemochromatosis-associated *HFE *genotypes is common in whites and blacks in Alabama [6-8], and *HJV *I222N and G320V were detected in an Alabama juvenile hemochromatosis index patient [3]. In addition, *HJV *I222N and G320V have been described in white adults with hemochromatosis phenotypes in other geographic areas [1,2,9].

## Methods

### General criteria for selection of study subjects

Performance of this study was approved by the Institutional Review Boards of the University of Alabama at Birmingham and Brookwood Medical Center. Unrelated white subjects were spouses of patients who attended a hematology and medical oncology outpatient clinic; hospital employees; and controls who participated in a sleep study. Unrelated control black subjects were spouses of patients who attended a hematology and medical oncology outpatient clinic; hospital employees; controls who participated in a sleep study; and controls who participated in a study of gestational diabetes mellitus. All subjects were adults (≥ 18 years old). No subject reported a previous diagnosis of hemochromatosis or iron overload. Serum transferrin saturation, serum ferritin concentration, or other indicators of iron metabolism were not measured in most study subjects.

### Polymerase chain reaction-restriction fragment length polymorphism (PCR-RFLP) analyses

Genomic DNA was isolated from blood buffy coat as described previously [6]. *HJV *I222N and G320V were genotyped by standard PCR-RFLP and agarose electrophoresis techniques. Exon 4 of the *HJV *locus was amplified using the primers HJEx4AF and HJEx4BR previously described [3]; this allowed for the amplification of the region containing both the I222N and G320V mutations of the *HJV *locus. Reaction conditions were as follows: 10 mM KCl, 10 mM (NH_4_)_2_SO_4_, 20 mM Tris-HCl (pH 8.8), 0.1% Triton X-100, 3.2 mM MgSO_4_, 200 μM dATP, 200 μM dCTP, 200 μM dGTP, 200 μM dTTP, 1 μM of each primer, 0.2 U/μL of Taq Polymerase. Amplification conditions on a Biometra UNO II thermocycler (LabRepCo/LabRepNet, Horsham, PA) consisted of an initial denaturing at 94°C for five minutes followed by thirty cycles of 94°C for 30 seconds, 60°C for 30 seconds and 72°C for 30 seconds.

Delineation of c.665T→A of the *HJV *exon 4 nucleotide sequence defining I222N was made utilizing the restriction endonuclease Bcc I (New England Biolabs, Beverly, MA), which recognizes the nucleotide c.665T of I222 as a restriction site, and not the nucleotide c.665A of N222. Similarly, the nucleotide polymorphism site c.959G→T defining the G320V polymorphism of the *HJV *exon 4 locus was determined by using the restriction endonuclease Ban I (New England Biolabs, Beverly, MA). Ban I recognizes the nucleotide c.959G of G320 as part of the restriction site sequence, but not the nucleotide c.959T of V320. DNA specimens from the parents of a white juvenile hemochromatosis index case were used as positive controls for *HJV *I222N and G320V [3].

### Statistical considerations

The data set consisted of observations on 241 Alabama white and 124 African American adults. Analysis for *HJV *I222N was performed in 240 whites and 124 African Americans. Analysis for *HJV *G320V was performed in 241 whites and 118 African Americans. Results are expressed as allele frequencies and 95% confidence intervals (CI). To estimate the CI for frequencies of *HJV *mutations that were not detected in the present subjects, we computed the allele frequency as the quotient of single minimal hypothetical value (= 0.01) and the number of chromosomes corresponding to the respective groups of subjects. Computations were performed with GB-STAT (v10.0; Dynamic Microsystems, Inc., Silver Spring, MD).

## Results

### Allele frequencies of *HJV *I222N and G320V

These results are displayed in Table 1. One of 240 white subjects was heterozygous for *HJV *I222N, but this mutation was not detected in African American subjects. *HJV *G320V was not detected in either white or African American subjects.

**Table 1:** *HJV *missense mutations in Alabama adult subjects from the general population.^1^

*HJV *mutation	I222N	G320V
Whites tested, n	240	241
Whites with mutation, n	1 (heterozygote)	0
Allele frequency (95% CI)	0.0021 (0, 0.0062)	0 (0, ~0.00006)
		
African Americans tested, n	124	118
African Americans with mutation, n	0	0
Allele frequency (95% CI)	0 (0, ~0.00012)	0 (0, ~0.00013)

^1^*HJV *mutations were detected using PCR-RFLP technique. DNA specimens from the parents of a white juvenile hemochromatosis index case were used as positive controls for *HJV *I222N and G320V [3]. CI = confidence interval.

### Characteristics of a *HJV *I222N heterozygote

Medical records of the white subject heterozygous for *HJV *I222N were available for review. This 29 year-old woman was also heterozygous for *HFE *C282Y. She reported that she had had two normal pregnancies and one spontaneous abortion. She has always experienced heavy menstrual flow, but she reported no other significant blood loss. She reported no blood donation. She reported that she eats a variety of meats, vegetables, and other foods; there was no history of supplemental iron ingestion. Her hemoglobin level was 12.8 g/dL, platelets 113,000/mm^3^, transferrin saturation 21%, and serum ferritin 23 ng/mL; marrow examination performed to evaluate thrombocytopenia revealed normal cellular morphology and normal iron stores.

## Discussion

A variety of *HJV *coding region mutations occur in homozygous or compound heterozygous configuration in persons with juvenile or adult-onset hemochromatosis phenotypes [1-5,9-11]. However, these types of *HJV*-associated hemochromatosis are collectively uncommon [1-3,11,12]. *HJV *mutations were also uncommon in control whites studied with dHPLC (denaturing high-performance liquid chromatography) [9]. In 200 Greek volunteer blood donors, none carried the *HJV *G320V mutation detectable by PCR-RFLP analysis, suggesting that the frequency of the G320V allele in the Greek population is lower than 0.004 [11]. In French control subjects, for example, only the *HJV *missense mutations L101P (two heterozygotes) and E302K (one heterozygote) were identified in 333 control subjects, representing an aggregate *HJV *mutation frequency of 0.0045 (95% CI: 0, 0.0096) [9]. The present results in Alabama white control subjects are consistent with these previous reports. Taken together, these observations confirm and extend previous observations that the individual or aggregate frequency of *HJV *coding region mutations is low in general populations of whites [9].

Heterozygosity for a *HJV *missense mutation was associated with increased severity of iron overload in nine of 310 French patients with hemochromatosis and *HFE *C282Y homozygosity who were studied with dHPLC [9]. In 48 white U.S. hemochromatosis patients with *HFE *C282Y homozygosity studied with dHPLC, no *HJV *coding region mutation was detected [5]. Thus, aggregate *HJV *coding region mutation frequencies in C282Y homozygotes with a hemochromatosis phenotype in France and the U.S. are similar (0.0148 vs. 0, respectively; p = 0.2716, Fisher exact test). The aggregate frequency of *HJV *coding region mutations was similar in French hemochromatosis patients with *HFE *C282Y homozygosity and in French control subjects (0.0145 vs. 0.0045, respectively; p = 0.0564, Fisher exact test), although the hemochromatosis patients with *HJV *coding region mutations had more severe iron overload, on the average, than did hemochromatosis patients without *HJV *coding region mutations [9].

Double heterozygosity for *HJV *I222N and *HFE *C282Y was not associated with evidence of increased iron absorption in the present case. Some persons with JH have common *HFE *genotypes, including C282Y heterozygosity, H63D heterozygosity or homozygosity, and S65C heterozygosity [13-18]. However, these *HFE *genotypes are infrequently associated with a severe hemochromatosis phenotype [6,7,19-25]. Further, sequencing *HFE *introns and exons in English and French Canadian JH cases did not reveal novel *HFE *mutations that could likely explain the development of iron overload [14,15]. In 310 C282Y homozygotes in France, *HJV *mutations were relatively common and were associated with greater severity of iron overload [9]. In 48 C282Y homozygotes in the U.S. who had severe iron overload phenotypes, no *HJV *coding region mutation was detected [5]. The frequency of H63D is significantly greater in persons with cardiomyopathy than in normal control subjects [26]. The brother of a Spanish JH proband had evidence of iron overload associated with H63D homozygosity and heterozygosity for a putative JH-associated Ch1q haplotype [17]. Although these reports are consistent with observations in another JH patient with cardiomyopathy who had *HFE *H63D [3,12], other persons with JH and cardiomyopathy did not have H63D [15]. Altogether, these reports indicate that further exploration of the potential role of variant *HJV *alleles in the clinical expression of iron overload with particular reference to *HFE *hemochromatosis is warranted [2], and that the frequency and biological significance of *HJV *alleles may vary among racial/ethnic groups.

Primary iron overload in African Americans is phenotypically and genetically heterogeneous [8]. Some patients have common *HFE *mutations, a common allele of the ferroportin gene (*FPN1 *Q248H), or heritable types of anemia [8,27,28], but the genetic basis of most cases remains unknown. *HJV *I222N and G320V were not detected in the present African American subjects. However, *HJV *coding region mutations were recently identified in two of 51 African American subjects with iron overload: exon 3, nt 205–207 ins GGA (ins G69) (frequency 0.0098); and exon 4, nt 929 C→G (A310G) (allele frequency 0.0196) [5]. The *HJV *alleles ins G69 and A310G were also identified in African American control subjects (allele frequencies 0.0038 and 0.0720, respectively) [5]. Taken together, these observations suggest that *HJV *coding region mutations are uncommon in African Americans, but may account for some cases of primary iron overload [5].

## Conclusions

*HJV *I222N and G320V are uncommon causes or modifiers of primary iron overload in adult whites and African Americans in Alabama. Double heterozygosity for *HJV *I222N and *HFE *C282Y may not promote increased iron absorption.

## Competing interests

The author(s) declare that they have no competing interests.

## Authors' contributions

JCB participated in conceiving the study, provided DNA specimens, performed statistical evaluation, and wrote part of the manuscript. CAR devised the PCR-RFLP assay, provided DNA specimens, participated in data collection, and wrote part of the manuscript. SN and SB performed testing on DNA specimens and participated in data collection. RTA participated in conceiving the study, provided DNA specimens, and wrote part of the manuscript. All authors approved the final version of the manuscript.

## Pre-publication history

The pre-publication history for this paper can be accessed here:



## References

[B1] Papanikolaou G, Samuels ME, Ludwig EH, MacDonald ML, Franchini PL, Dube MP, Andres L, MacFarlane J, Sakellaropoulos N, Politou M, Nemeth E, Thompson J, Risler JK, Zaborowska C, Babakaiff R, Radomski CC, Pape TD, Davidas O, Christakis J, Brissot P, Lockitch G, Ganz T, Hayden MR, Goldberg YP (2004). Mutations in HFE2 cause iron overload in chromosome 1q-linked juvenile hemochromatosis. Nat Genet.

[B2] Lanzara C, Roetto A, Daraio F, Rivard S, Ficarella R, Simard H, Cox TM, Cazzola M, Piperno A, Gimenez-Roqueplo AP, Grammatico P, Volinia S, Gasparini P, Camaschella C (2004). Spectrum of hemojuvelin gene mutations in 1q-linked juvenile hemochromatosis. Blood.

[B3] Lee PL, Beutler E, Rao SV, Barton JC (2004). Genetic abnormalities and juvenile hemochromatosis: mutations of the HJV gene encoding hemojuvelin. Blood.

[B4] Robson KJ, Merryweather-Clarke AT, Cadet E, Viprakasit V, Zaahl MG, Pointon JJ, Weatherall DJ, Rochette J (2004). Recent advances in understanding haemochromatosis: a transition state. J Med Genet.

[B5] Lee PL, Barton JC, Brandhagen D, Beutler E (2004). Hemojuvelin (HJV) mutations in persons of European, African-American and Asian ancestry with adult onset haemochromatosis. Br J Haematol.

[B6] Barton JC, Shih WW, Sawada-Hirai R, Acton RT, Harmon L, Rivers C, Rothenberg BE (1997). Genetic and clinical description of hemochromatosis probands and heterozygotes: evidence that multiple genes linked to the major histocompatibility complex are responsible for hemochromatosis. Blood Cells Mol Dis.

[B7] Barton JC, Sawada-Hirai R, Rothenberg BE, Acton RT (1999). Two novel missense mutations of the HFE gene (I105T and G93R) and identification of the S65C mutation in Alabama hemochromatosis probands. Blood Cells Mol Dis.

[B8] Barton JC, Acton RT, Rivers CA, Bertoli LF, Gelbart T, West C, Beutler E (2003). Genotypic and phenotypic heterogeneity of African Americans with primary iron overload. Blood Cells Mol Dis.

[B9] Le Gac G, Scotet V, Ka C, Gourlaouen I, Bryckaert L, Jacolot S, Mura C, Ferec C (2004). The recently identified type 2A juvenile haemochromatosis gene (HJV), a second candidate modifier of the C282Y homozygous phenotype. Hum Mol Genet.

[B10] Huang FW, Rubio-Aliaga I, Kushner JP, Andrews NC, Fleming MD (2004). Identification of a novel mutation (C321X) in HJV. Blood.

[B11] Pissia M, Polonifi K, Politou M, Lilakos K, Sakellaropoulos N, Papanikolaou G (2004). Prevalence of the G320V mutation of the HJV gene, associated with juvenile hemochromatosis, in Greece. Haematologica.

[B12] Barton JC, Rao SV, Pereira NM, Gelbart T, Beutler E, Rivers CA, Acton RT (2002). Juvenile hemochromatosis in the southeastern United States: a report of seven cases in two kinships. Blood Cells Mol Dis.

[B13] Kaltwasser JP, Barton JC and Edwards CQ (2000). Juvenile hemochromatosis. Hemochromatosis  Genetics, pathophysiology, diagnosis, and treatment.

[B14] Rivard SR, Mura C, Simard H, Simard R, Grimard D, Le Gac G, Raguenes O, Ferec C, De Braekeleer M (2000). Clinical and molecular aspects of juvenile hemochromatosis in Saguenay-Lac-Saint-Jean (Quebec, canada). Blood Cells Mol Dis.

[B15] Kelly AL, Rhodes DA, Roland JM, Schofield P, Cox TM (1998). Hereditary juvenile haemochromatosis: a genetically heterogeneous life-threatening iron-storage disease. QJM.

[B16] Varkonyi J, Kaltwasser JP, Seidl C, Kollai G, Andrikovics H, Tordai A (2000). A case of non-HFE juvenile haemochromatosis presenting with adrenocortical insufficiency. Br J Haematol.

[B17] Montes-Cano M, Gonzalez-Escribano MF, Aguilar J, Nunez-Roldan A (2002). Juvenile hemochromatosis in a Spanish family. Blood Cells Mol Dis.

[B18] Roetto A, Totaro A, Cazzola M, Cicilano M, Bosio S, D'Ascola G, Carella M, Zelante L, Kelly AL, Cox TM, Gasparini P, Camaschella C (1999). Juvenile hemochromatosis locus maps to chromosome 1q. Am J Hum Genet.

[B19] Feder JN, Gnirke A, Thomas W, Tsuchihashi Z, Ruddy DA, Basava A, Dormishian F, Domingo RJ, Ellis MC, Fullan A, Hinton LM, Jones NL, Kimmel BE, Kronmal GS, Lauer P, Lee VK, Loeb DB, Mapa FA, McClelland E, Meyer NC, Mintier GA, Moeller N, Moore T, Morikang E, Wolff RK (1996). A novel MHC class I-like gene is mutated in patients with hereditary haemochromatosis. Nat Genet.

[B20] Beutler E, Gelbart T, West C, Lee P, Adams M, Blackstone R, Pockros P, Kosty M, Venditti CP, Phatak PD, Seese NK, Chorney KA, Ten Elshof AE, Gerhard GS, Chorney M (1996). Mutation analysis in hereditary hemochromatosis. Blood Cells Mol Dis.

[B21] Beutler E (1997). The significance of the 187G (H63D) mutation in hemochromatosis. Am J Hum Genet.

[B22] Adams PC, Chakrabarti S (1998). Genotypic/phenotypic correlations in genetic hemochromatosis: evolution of diagnostic criteria. Gastroenterology.

[B23] Mura C, Raguenes O, Ferec C (1999). HFE mutations analysis in 711 hemochromatosis probands: evidence for S65C implication in mild form of hemochromatosis. Blood.

[B24] Rossi E, Henderson S, Chin CY, Olynyk J, Beilby JP, Reed WD, Jeffrey GP (1999). Genotyping as a diagnostic aid in genetic haemochromatosis. J Gastroenterol Hepatol.

[B25] Gochee PA, Powell LW, Cullen DJ, Du SD, Rossi E, Olynyk JK (2002). A population-based study of the biochemical and clinical expression of the H63D hemochromatosis mutation. Gastroenterology.

[B26] Camaschella C, Roetto A, Cali A, De Gobbi M, Garozzo G, Carella M, Majorano N, Totaro A, Gasparini P (2000). The gene TFR2 is mutated in a new type of haemochromatosis mapping to 7q22. Nat Genet.

[B27] Beutler E, Felitti V, Gelbart T, Ho N (2000). The effect of HFE genotypes on measurements of iron overload in patients attending a health appraisal clinic. Ann Intern Med.

[B28] Beutler E, Barton JC, Felitti VJ, Gelbart T, West C, Lee PL, Waalen J, Vulpe C (2003). Ferroportin 1 (SCL40A1) variant associated with iron overload in African-Americans. Blood Cells Mol Dis.

